# Supplementary material for: A broadly distributed toxin family mediates contact-dependent antagonism between gram-positive bacteria
Source: eLife. 2017 Jul 11;6:e26938. doi: 10.7554/eLife.26938 (PMC5555719; doi:10.7554/eLife.26938)
Supplement: Supplementary file 1. — DOI: http://dx.doi.org/10.7554/eLife.26938.020 [file elife-26938-supp1.docx]

**Additional File 1. Strains used in this study.**

| Organism | Genotype | Reference |
| --- | --- | --- |
| *S. intermedius* ATCC 27335 | wild-type | (Whiley and Beighton, 1991) |
| *S. intermedius* B196 | wild-type | (Olson et al., 2013) |
|  | SIR_0115 ::*spec* | This study |
|  | Δ*essC* ::*spec* | This study |
|  | Δ*telB* ::*spec* | This study |
|  | Δ*telC* ::*spec* | This study |
|  | Δ*telB* Δ*tipB* ::*kan* | This study |
|  | Δ*telC* Δ*tipC* ::*kan* | This study |
|  | Δ*wxgC* ::*kan* | This study |
| *S. pyogenes* 5005 | wild-type | (Lukomski et al., 2000) |
| *S. aureus* USA300 | wild-type | (Diep et al., 2006) |
| *E. faecalis* OG1RF | wild-type | (Xu et al., 1998) |
| *E. coli* MG1655 | wild-type | (Blattner et al., 1997) |
| *P. aeruginosa*  PAO1 | wild-type | (Stover et al., 2000) |
| *B. thailandensis*  E264 | wild-type | (Kim et al., 2005) |
| *B. fragilis*  NCTC9343 | wild-type | (Cerdeno-Tarraga et al., 2005) |
| *S. cerevisiae* BY4742 | *MAT*α *his3*Δ*1 leu2*Δ*0 lys2*Δ*0 ura3*Δ*0* | (Ho et al., 2002) |
| *E. coli* DH5α | F^-^ endA1 glnV44 thi-1 recA1 relA1 gyrA96 deoR nupG Φ80d*lacZ*ΔM15 Δ(*lacZYA-argF*)U169, hsdR17(r_K_^-^ m_K_^+^), λ– | Novagen |
| *E. coli* BL21 (DE3) pLysS | F^-^ ompT gal dcm lon hsdS_B_(r_B_^-^ m_B_^-^) λ(DE3) pLysS(cm^R^) | Novagen |
| *E. coli* BTH101 | F^-^, *cya-99*, *araD139*, *galE15*, *galK16*, *rpsL1* (str^R^), *hsdR2*, *mcrA1*, *mcrB1* | Euromedex |

References:

Blattner, F.R., Plunkett, G., 3rd, Bloch, C.A., Perna, N.T., Burland, V., Riley, M., Collado-Vides, J., Glasner, J.D., Rode, C.K., Mayhew, G.F.*, et al.* (1997). The complete genome sequence of Escherichia coli K-12. Science *277*, 1453-1462.

Cerdeno-Tarraga, A.M., Patrick, S., Crossman, L.C., Blakely, G., Abratt, V., Lennard, N., Poxton, I., Duerden, B., Harris, B., Quail, M.A.*, et al.* (2005). Extensive DNA inversions in the B. fragilis genome control variable gene expression. Science *307*, 1463-1465.

Diep, B.A., Gill, S.R., Chang, R.F., Phan, T.H., Chen, J.H., Davidson, M.G., Lin, F., Lin, J., Carleton, H.A., Mongodin, E.F.*, et al.* (2006). Complete genome sequence of USA300, an epidemic clone of community-acquired meticillin-resistant Staphylococcus aureus. Lancet *367*, 731-739.

Ho, Y., Gruhler, A., Heilbut, A., Bader, G.D., Moore, L., Adams, S.L., Millar, A., Taylor, P., Bennett, K., Boutilier, K.*, et al.* (2002). Systematic identification of protein complexes in Saccharomyces cerevisiae by mass spectrometry. Nature *415*, 180-183.

Kim, H.S., Schell, M.A., Yu, Y., Ulrich, R.L., Sarria, S.H., Nierman, W.C., and DeShazer, D. (2005). Bacterial genome adaptation to niches: divergence of the potential virulence genes in three Burkholderia species of different survival strategies. BMC genomics *6*, 174.

Lukomski, S., Hoe, N.P., Abdi, I., Rurangirwa, J., Kordari, P., Liu, M., Dou, S.J., Adams, G.G., and Musser, J.M. (2000). Nonpolar inactivation of the hypervariable streptococcal inhibitor of complement gene (sic) in serotype M1 Streptococcus pyogenes significantly decreases mouse mucosal colonization. Infection and immunity *68*, 535-542.

Olson, A.B., Kent, H., Sibley, C.D., Grinwis, M.E., Mabon, P., Ouellette, C., Tyson, S., Graham, M., Tyler, S.D., Van Domselaar, G.*, et al.* (2013). Phylogenetic relationship and virulence inference of Streptococcus Anginosus Group: curated annotation and whole-genome comparative analysis support distinct species designation. BMC genomics *14*, 895.

Stover, C.K., Pham, X.Q., Erwin, A.L., Mizoguchi, S.D., Warrener, P., Hickey, M.J., Brinkman, F.S., Hufnagle, W.O., Kowalik, D.J., Lagrou, M.*, et al.* (2000). Complete genome sequence of Pseudomonas aeruginosa PA01, an opportunistic pathogen. Nature *406*, 959-964.

Whiley, R.A., and Beighton, D. (1991). Emended descriptions and recognition of Streptococcus constellatus, Streptococcus intermedius, and Streptococcus anginosus as distinct species. International journal of systematic bacteriology *41*, 1-5.

Xu, Y., Murray, B.E., and Weinstock, G.M. (1998). A cluster of genes involved in polysaccharide biosynthesis from Enterococcus faecalis OG1RF. Infection and immunity *66*, 4313-4323.
