# Supplementary material for: A broadly distributed toxin family mediates contact-dependent antagonism between gram-positive bacteria
Source: eLife. 2017 Jul 11;6:e26938. doi: 10.7554/eLife.26938 (PMC5555719; doi:10.7554/eLife.26938)
Supplement: Supplementary file 2. — DOI: http://dx.doi.org/10.7554/eLife.26938.021 [file elife-26938-supp2.docx]

**Additional File 2. Plasmids used in this study.**

| Plasmid | Relevant features | Reference |
| --- | --- | --- |
| pSCrhaB2 | Expression vector with *rhaB*, Tmp^R^, rhamnose inducible | (Cardona and Valvano, 2005) |
| pETDuet-1 | Co-expression vector with *lacI*, T7 promoter, N-terminal His_6_ tag in MCS-1, Amp^R^ | Novagen |
| pET29b | Expression vector with *lacI*, T7 promoter, C-terminal His_6_ tag, Kan^R^ | Novagen |
| pEPSA5 | Expression vector with *xylR*, T5X promoter, Amp^R^, Chlor^R^, xylose-inducible | (Forsyth et al., 2002) |
| pDL277 | *Streptococcus*-*E.coli* shuttle vector, Spec^R^ | (Aspiras et al., 2000) |
| pCM190 | *S. cerevisiae* expression vector with *tetO*-tTA, doxycycline repressible | (Gari et al., 1997) |
| pKT25 | B2H expression vector with *plac*, Kan^R^, N-terminal fusion to T25 fragment of CyaA | Euromedex |
| pKNT25 | B2H expression vector with *plac*, Kan^R^, C-terminal fusion to T25 fragment of CyaA | Euromedex |
| pUT18 | B2H expression vector with *plac*, Amp^R^, N-terminal fusion to T18 fragment of CyaA | Euromedex |
| pUT18C | B2H expression vector with *plac*, Amp^R^, C-terminal fusion to T18 fragment of CyaA | Euromedex |
| pSCrhaB2::PA2702 | *E. coli* expression vector for *tse2* | (Silverman et al., 2013) |
| pSCrhaB2::SIR_0179_543-743_H661A | *E. coli* expression vector for *telB_543-743_* H661A point mutant | This study |
| pSCrhaB2::SIR_0179_543-743_ R626A_H661A | *E. coli* expression vector for *telB_543-743_* R626A, H661A double mutant | This study |
| pSCrhaB2::PA0093_282-430 | *E. coli* expression vector for *tse6_282-430_* | (Whitney et al., 2014) |
| pSCrhaB2::SIR_0169_303-536 | *E. coli* expression vector for *telA_303-536_* | This study |
| pETDuet-1::SIR_0170 | *E. coli* expression vector for *tipA* | This study |
| pETDuet-1::SIR_0180 | *E. coli* expression vector for *tipB* | This study |
| pDL277::P96_SIR_1489_202-552 | *S. intermedius* expression vector for *telC_202-552_* with P96 promoter and start codon | This study |
| pDL277::P96_ssSIR_1489_202-552 | *S. intermedius* expression vector for *telC_202-552_* fused to a sec signal sequence with P96 promoter | This study |
| pDL277::P96_ssSIR_1489_202-552–SIR_1488 | *S. intermedius* expression vector for *telC_202-552_* fused to a sec signal sequence and *tipC* with P96 promoter | This study |
| pDL277::P96_ssSIR_1489_202-552_D401A | *S. intermedius* expression vector for *telC_202-552_* D401A fused to a sec signal sequence with P96 promoter | This study |
| pDL277::P96_ssSIR_1489_202-552_D455A | *S. intermedius* expression vector for *telC_202-552_* D455A fused to a sec signal sequence with P96 promoter | This study |
| pDL277::P96_ssSIR_1489__202-552_D459A | *S. intermedius* expression vector for *telC_202-552_* D459A fused to a sec signal sequence with P96 promoter | This study |
| pEPSA5::SIR_1489_202-552 | *S. aureus* expression vector for *telC_202-552_* | This study |
| pEPSA5::ssSIR_1489_202-552 | *S. aureus* expression vector for *ss-telC_202-552_* | This study |
| pEPSA5::ssSIR_1489_202-552_D401A | *S. aureus* expression vector for *ss-telC_202-552_* D401A | This study |
| pETDuet-1:: SIR_1489 | *E. coli* expression vector for *telC* | This study |
| pETDuet-1:: SIR_1489_202-552 | *E. coli* expression vector for *telC_202-552_* | This study |
| pETDuet-1:: SIR_1488_23-204 | *E. coli* expression vector for *tipC_202-552_* | This study |
| pKT25::zip | Control B2H expression vector | Euromedex |
| pUT18C::zip | Control B2H expression vector | Euromedex |
| pKT25:: SIR_1488_23-204 | B2H expression vector for *tipC* | This study |
| pUT18::SIR_1489 | B2H expression vector for *telC* | This study |
| pUT18::SIR_1489_222-552 | B2H expression vector for *telC_222-552_* | This study |
| pUT18C::SIR_1489_1-222 | B2H expression vector for *telC_1-222_* | This study |
| pUT18C::SIR_0169_1-224 | B2H expression vector for *telA_1-224_* | This study |
| pUT18C::SIR_0179_1-279 | B2H expression vector for *telB_1-279_* | This study |
| pKNT25::SIR_166.1 | B2H expression vector for *wxgA* | This study |
| pKNT25::SIR_177 | B2H expression vector for *wxgB* | This study |
| pKNT25::SIR_1491 | B2H expression vector for *wxgC* | This study |
| pET29b::SIR_0115 ::*spec* | For inserting spectinomycin cassette into neutral chromosomal site | This study |
| pET29b::∆SIR_0175 ::*spec* | For generating *essC* mutant | This study |
| pET29b::∆SIR_0179 ::*spec* | For generating *telB* mutant | This study |
| pET29b::∆SIR_1489 ::*spec* | For generating *telC* mutant | This study |
| pET29b::∆SIR_0179-0190 ::*kan* | For generating *telB*-*tipB* mutant. Also knocks out downstream poly-immunity cluster. | This study |
| pET29b::∆SIR_1486-1489 ::*kan* | For generating *telC*-*tipC* mutant. Also knocks out downstream toxin-immunity repeat. | This study |
| pET29b::∆SIR_1491 ::*kan* | For generating *wxgC* mutant | This study |
| pET29b::SIR_0177_CV | *E. coli* expression vector for *wxgB* fused to a C-terminal VSV-G tag | This study |
| pET29b::SIR_1491_CV | *E. coli* expression vector for *wxgC* fused to a C-terminal VSV-G tag | This study |

References:

Aspiras, M.B., Kazmerzak, K.M., Kolenbrander, P.E., McNab, R., Hardegen, N., and Jenkinson, H.F. (2000). Expression of green fluorescent protein in Streptococcus gordonii DL1 and its use as a species-specific marker in coadhesion with Streptococcus oralis 34 in saliva-conditioned biofilms in vitro. Applied and environmental microbiology *66*, 4074-4083.

Cardona, S.T., and Valvano, M.A. (2005). An expression vector containing a rhamnose-inducible promoter provides tightly regulated gene expression in Burkholderia cenocepacia. Plasmid *54*, 219-228.

Forsyth, R.A., Haselbeck, R.J., Ohlsen, K.L., Yamamoto, R.T., Xu, H., Trawick, J.D., Wall, D., Wang, L., Brown-Driver, V., Froelich, J.M.*, et al.* (2002). A genome-wide strategy for the identification of essential genes in Staphylococcus aureus. Mol Microbiol *43*, 1387-1400.

Gari, E., Piedrafita, L., Aldea, M., and Herrero, E. (1997). A set of vectors with a tetracycline-regulatable promoter system for modulated gene expression in Saccharomyces cerevisiae. Yeast *13*, 837-848.

Silverman, J.M., Agnello, D.M., Zheng, H., Andrews, B.T., Li, M., Catalano, C.E., Gonen, T., and Mougous, J.D. (2013). Haemolysin Coregulated Protein Is an Exported Receptor and Chaperone of Type VI Secretion Substrates. Molecular cell *51*, 584-593.

Whitney, J.C., Beck, C.M., Goo, Y.A., Russell, A.B., Harding, B.N., De Leon, J.A., Cunningham, D.A., Tran, B.Q., Low, D.A., Goodlett, D.R.*, et al.* (2014). Genetically distinct pathways guide effector export through the type VI secretion system. Molecular microbiology *92*, 529-542.
